# Nuclear Magnetic Resonance Microimaging for the Qualitative Assessment of Root Canal Treatment: An Ex Vivo Preliminary Study

**DOI:** 10.3390/diagnostics11061012

**Published:** 2021-06-01

**Authors:** Silvia Capuani, Gianluca Gambarini, Renzo Guarnieri, Giulia Di Pietro, Luca Testarelli, Dario Di Nardo

**Affiliations:** 1 CNR ISC c/o Physics Department, Sapienza University of Rome, 00185 Rome, Italy; silvia.capuani@isc.cnr.it (S.C.); giulia.dipietro.dpg@gmail.com (G.D.P.); 2 Museo Storico Della Fisica e Centro Studi e Ricerche Enrico Fermi, 00185 Rome, Italy; 3 Department of Oral and Maxillo Facial Sciences, Sapienza Università di Roma, 00161 Rome, Italy; Gianluca.gambarini@uniroma1.it (G.G.); renzoguarnieri@gmail.com (R.G.); dario.dinardo@uniroma1.it (D.D.N.)

**Keywords:** cone beam computed tomography, endodontic treatment, magnetic resonance imaging, tooth micro-imaging, MRI multi-parametric maps

## Abstract

Aim: To assess the potential ability of nuclear magnetic resonance micro-imaging (mMRI) to visualize and identify soft tissue debris and unfilled spaces inside radicular canals in endodontic treated extracted teeth, for understanding the causes of treatment failure. Toward this goal, multi-parametric mMRI and cone beam computed tomography (CBCT) were compared. Methodology: A non-recoverable root treated human tooth was extracted due to endodontic failure and excessive mobility. It was examined with both CBCT and mMRI: CBCT was performed with 0.125 mm voxel size (GXCB-500, Kavo-Gendex, Brea, CA, USA) and mMRI was performed with a spectrometer operating at 9.4T magnetic field (Bruker Avance-400, Bruker, Billerica, MA, USA). The mMRI images were obtained with a microimaging probe. Relaxation times (T1 and T2) and diffusion-weighted acquisition sequences were used to obtain multi-parametric maps of the extracted tooth (slice thickness of 200 µm and in plane resolution of 30 × 30 µm^2^). Results: T1 and T2 maps identified unfilled spaces around and close to Gutta-percha cones instead of CBCT images that were not able to highlight this aspect. T1, T2 and apparent diffusion coefficient (ADC) assumed different values in dentine and in voids, characterized by different dimensions. Moreover, they were able to discriminate between infiltrations of water only and deposits of biological material. Because Gutta-percha cones are constituted of hard, non-porous material, they do not provide a signal and in mMRI images appear as zones of noise. Conclusions: Unlike the CBCT exam, mMRI can detect soft tissue debris and unfilled spaces inside radicular canals. Therefore, this in vitro study showed the potential of mMRI to evaluate the quality of the root canal treatment, suggesting its potential benefit in determining the causes of endodontic failure, without the use of ionizing radiation.

## 1. Introduction

The rationale for endodontic treatment is to eradicate infection and prevent microorganisms from infecting or re-infecting the root and its peri-radicular tissues by achieving a three-dimensional filling of the cleaned and shaped root canal system. Failure to achieve these goals can result in continued presence of inflammation and infection, recontamination, and negative outcome of the endodontic treatment [1].

During clinical procedures, canal obturation quality is usually determined using bi-dimensional periapical radiographs. Experimentally, destructive methods such as histological analysis are employed. On the other hand, volumetric acquisitions provided by computed tomography (CT) or nuclear magnetic resonance (NMR) allow the study of three-dimensional root structures without the destruction of the specimen. Song et al. [2] examined the clinical causes of failure of nearly 500 previous endodontic treatments by an inspection of the root apex and resected root surface at 26× magnification during endodontic microsurgery. The most common possible cause of failure was the presence of a leakage around the canal filling material (30.4%), followed by a missing canal (19.7%), and underfilling (14.2%). Voids and canal underfilling can be present in 2D or 3D radiographic images and are perceived as an incomplete treatment. However, traditional radiographic methods cannot show exactly if the failure is due to remaining voids, or if it originates from iatrogenic errors performed by the operator during the treatment. In the first case, the goal of adequate debridement and disinfection could be achieved, but iatrogenic errors often could definitively compromise the outcome.

Leakage around the filling material is more difficult to evaluate by cone beam computed tomography (CBCT): it is possible to visualize a “missed treatment”, a portion of endodontic space that has not been obturated, but the image quality is often distorted by the radiodensity of the Gutta-percha cone. [3] Hoen and Pink [4] evaluated 1100 root canal failures and showed that 42% of them presented empty canal spaces. This appears as radiolucency (“unfilled areas”) inside the filled root canal system both in transversal and longitudinal cuts. Such missed treatment is likely to be related to poor cleaning and shaping procedures: remnants of pulpal soft tissues, which are potentially infected, are left inside the canal space, and inorganic debris also produced by instrumentation can be present. Voids into the canal system could be also due to an insufficient three-dimensional filling caused by an inadequate technique.

Recent micro-CT studies [5,6] showed inorganic debris left inside the canal after root canal preparation, which could also be defined as missed treatment. Unfortunately, with micro-CT we cannot detect soft tissues left inside prepared root canals and this type of exam cannot be performed clinically. When a missed treatment is shown by “in vitro” studies performed by non-invasive methodologies (CBCT or micro-CT), a void due to inadequate root canal filling procedure is visible, but the nature of what is left inside this “void” can be assessed only by histology, which is a destructive methodology and cannot be used clinically.

These problems could be overcome by a technique that would enable the visualization of remnants of soft pulp tissues and unfilled interstices inside root canal space after endodontic procedures. Magnetic resonance imaging (MRI) has become the primary diagnostic investigation for many clinical problems, due to its capability to differentiate between soft tissues and hard tissues, without the use of ionizing radiation. Currently, MRI investigation has been successfully used in dentistry [7,8] and the first applications in endodontics are being developed [9,10]. Clinical MRI, characterized by magnetic field strength from 0.5 T to 3.0 T and by magnetic field gradient strength of up to 80 mT/m, provides images with a linear resolution of about 1 mm in soft tissues, which worsens in porous solid tissues such as cancellous bone. However, high-field magnetic resonance scanners (with a magnetic field higher than 7 T) with high-performance magnetic field gradients (up to 2000 mT/m) enable MR micro imaging (mMRI) investigations, providing images with a linear resolution less than 100 microns [10,11,12,13]. Currently, the technology to achieve mMRI is available in laboratory NMR spectrometers, which allow us to analyze small samples and are therefore suitable for in vitro experiments.

The possibility of clearly identifying the causes which leaded to an endodontic failure could help the practitioner to perform a correct therapeutic plan and avoid starting useless treatments due to a wrong diagnosis. MRI can detect and differentiate pathologic tissues from healthy ones and this kind of information is fundamental when the decision-making process has to be performed: endodontic re-treatment is usually a challenging operation which could easily fail and make the tooth irrecoverable anymore, so whether or not it is necessary should be assessed prior to start on the basis of strong evidence given by a reliable diagnostic tool [10]. The goal of the present study was to describe the use of multiparametric mMRI, as a non-invasive testing methodology, to identify voids, debris, and soft tissues left inside a treated root canal. The method that we tested in vitro is based on obtaining multiparametric maps, representing the spatial distribution of MRI parameters: spin lattice relaxation time (T1), spin-spin relaxation time (T2), and apparent diffusion coefficient (ADC), which assume different values in more or less confined water present in residual pulp tissues, were adopted [14]. The root of the canine was extracted because the tooth was affected by periapical lesion, excessive mobility and its prosthetic crown was lost and not recoverable. In this case, the most likely cause of the periapical lucency was that some areas of the root canal were not cleaned properly or filled with Gutta-percha. CBCT was used as a consolidated methodology in endodontics to compare, validate and discuss mMRI results.

## 2. Materials and Methods

In this preliminary study, the experimental design includes the achievement of multiparametric mMRI images of the same endodontically treated tooth and the obtainment of CBCT images to validate and compare mMRI.

### 2.1. Materials

A non-recoverable endodontically treated inferior canine which had been extracted was selected for the study. A short metallic post was removed during the extraction procedure. The root was filled with cold Gutta-percha sealing technique. Immediately after the extraction the tooth was kept in a NaCL 0.9% solution at 4 °C for two days. Then, it was positioned in an NMR glass capillary with its longest axis along the main direction of the static magnetic field. The capillary was filled with tap water and the sample temperature was fixed to 20 °C.

### 2.2. Magnetic Resonance Imaging

MRI experiments were performed using a Bruker Avance-400 (Bruker, Billerica, MA, USA) high-resolution spectrometer operating at 9.4 T with a micro-imaging probe (10 mm internal diameter), equipped with a gradient unit characterized by a maximum gradient strength of 1200 mT/m and a rise time of 100 μs. XWINNMR^®^ (Bruker, Billerica, MA, USA) and ParaVision^®^ 3.0 (Bruker, Billerica, MA, USA) software were employed for data acquisition and analysis.

To obtain T2-weighted images in transversal and axial view, multi slice multi echo (MSME) imaging sequences were adopted with the following parameters: repetition time (TR) = 2000 ms; field of view (FOV) = 15 × 15 mm^2^; image matrix 256 × 256; slice thickness (STH) = 0.3 mm; number of slices, 24; voxel dimension 58 × 58 × 300 µm^3^; number of average scans (NS) = 32; acquisitions were performed at 24 different echo times (TE), ranging from 2.3 ms to 55.2 ms. Moreover, MSME imaging sequences with the following parameters were performed to obtain transversal and axial T1-weighted images: TE = 1.8 ms, FOV = 15 × 15 mm^2^, image matrix 256 × 256, STH=0.3mm, number of slices, 24; voxel dimension 58 × 58 × 300 µm^3^, NS = 32; acquisitions were performed using TR = 2000 ms, 900 ms, 500 ms, 300 ms and 150 ms. Finally, an axial pulse-gradient stimulated-echo (PGSTE) imaging sequence was employed (TE/TR = 11.3/2500 ms, FOV = 7 mm, matrix 128 × 128, 10 slices, STH = 0.6 mm, NS = 64, diffusion time Δ = 60 ms, diffusion gradient duration δ = 3 ms) using three effective b-values: 167, 1467 and 3167 s/mm^2^ with diffusion gradient direction perpendicular to the main axis of the tooth, in order to obtain diffusion weighted (DW) images with voxel dimension 58 × 58 × 600 µm^3^. The b-value, that selects the molecular diffusion regime, is related to the aforementioned parameters through the known relation: b = γ^2^g^2^δ^2^(Δ − δ/3) [15], where g is the diffusion gradient strength, ™ is the duration of the gradient pulse and Δ, that is the time between the leading edges of the pair of diffusion gradient pulses, is the diffusion time [16]. 

### 2.3. Images Analysis 

The acquired T1, T2 and apparent diffusion coefficient (ADC) weighted images were exported in DICOM format to a post-processing workstation (Z800, Hewlett Packard, San Jose, CA, USA). Fitting procedures in each voxel and image post-processing procedures were performed to obtain T1, T2 and ADC color-coded maps. T1, T2 and ADC values were calculated in regions of interest (ROI) which were identified on the maps by one skilled endodontist (G.G.) and one MRI-physicist (S.C), with more than 20 years of experience in endodontics and MRI, respectively. 

In order to obtain T2-maps, the follow spin echo (SE) decay function:

S(TE) = S(0) × exp(−TE/T2) + c(1)

was fitted to experimental data in each image voxel, were S(TE) and S(0) are the MRI signal at echo time TE and TE = 0, respectively, c is a constant to take into account for the noise floor. 

T1-maps were obtained using the function:

S(TR) = S(0) × [1 − exp(−TR/T1)](2)

were S(TR) is the MRI signal as a function of the repetition time TR.

Finally, ADC map were obtained using the function:

S(b) = S(0) × exp(−b × ADC) + c(3)

were S(b) and S(0) are the MRI signal at a given b-value and at b = 0, respectively.

### 2.4. Cone Beam Computed Tomography 

After mMRI investigation, X-ray imaging was performed using a CBCT (KaVo Gendex Dental Systems CB500, Hatfield, Pennsylvania, US) with the following volumetric acquisition protocol: 5 mA, 120 kV, STH = 0.125 mm, FOV = 8.9 × 3.0 cm, voxel dimension 0.125 × 0.125 × 0.125 mm^3^. Images were processed by iCATVision™ (Imaging Sciences International, Hatfield, Pennsylvania).

## 3. Results

**Figure 1:**
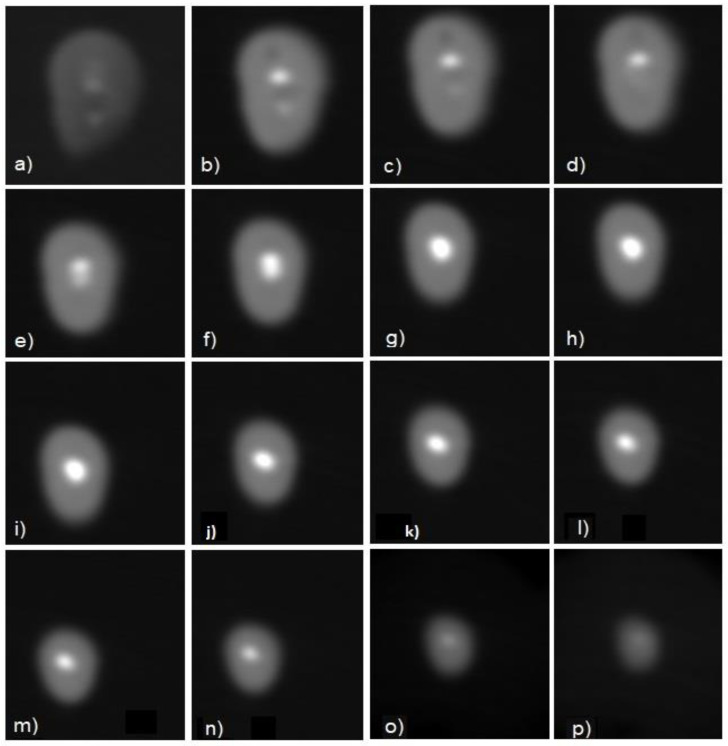
Axial CBCT acquisitions (0.125 mm thickness) of middle (**a**–**h**) and apical third (**i**–**p**). The presence of two Gutta-percha cones is appreciable at the beginning of the middle third (**a**–**d**). Apically, the presence of a single cone can be assessed by a decrescent radiodensity, barely visible at the end of the root (**p**). Boundaries between the Gutta-percha cone and the dental tissue are blurry due to scattering which cannot allow the operator to clearly define the presence of voids.

CBCT confirmed the presence of an incomplete root canal filling: Gutta-percha in the coronal third appeared partially removed, probably due to the previous presence of the post. Middle third appeared filled in the three dimensions, while apical filling is incomplete and appears 1.5 mm short from the anatomic apex. It was not possible to visualize pulp tissue or root canal voids in the apical last millimeters. Even with a 0.125 × 0.125 × 0.125 mm^3^ resolution, it was not possible to exactly determine where the apical foramen or constriction are located. The last apical 1–2 mm may be filled by debris, being radiopaque as dentine. As expected, the resolution of the CBCT acquisition was not sufficient to clearly visualize the apical third, which appears blurry, and only the absence of Gutta-percha in the very last millimeters can be recognized by a reduction of radiodensity (Figure 1).

**Figure 2:**
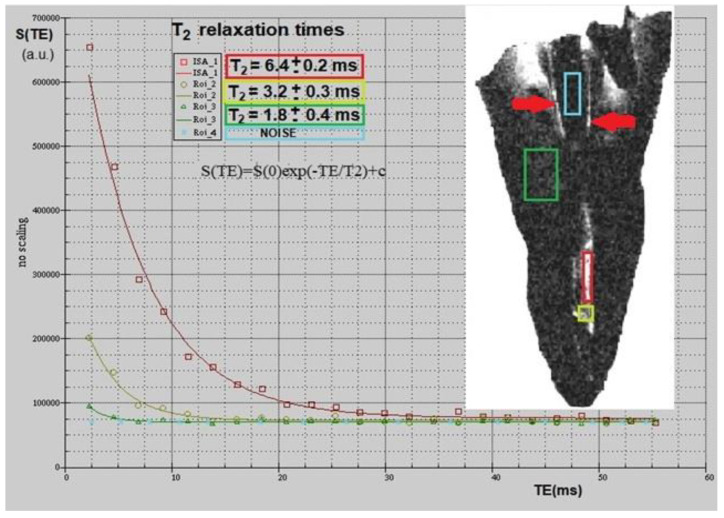
T2-weighted mMRI of tooth’s transverse section (white insert) with the different ROIs from which the data were extracted: light blue, Gutta-percha; green, dentine; red and yellow, voids and underfilled canals. The graph shows the experimental data decay vs. echo time (TE) in different tooth ROIs and the resulting fit function. From the fit of the function to data, T2 values were obtained in different regions of the tooth. Red arrows indicate spaces filled with water between dentine and Gutta-percha.

**Figure 3:**
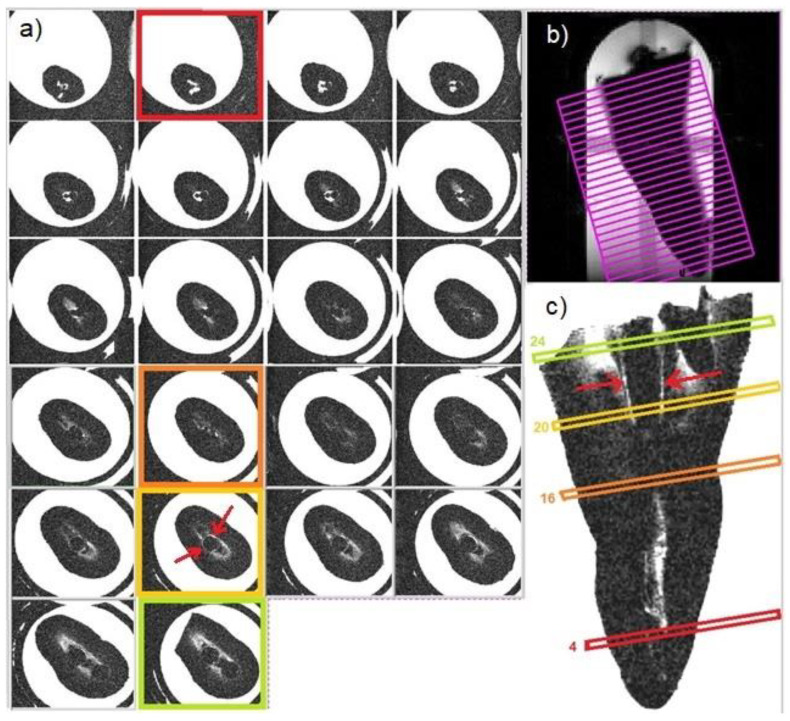
Axial view obtained by mMRI (**a**): slices correspond to the violet slices of the caption (**b**). In the transversal view (**c**), slices are highlighted with different colors, corresponding to the colored squares in caption (**a**). Red arrows in the transversal tooth view and axial yellow marked slice indicate spaces filled with water between dentine and Gutta-percha, confirming the presence of a void.

**Figure 4:**
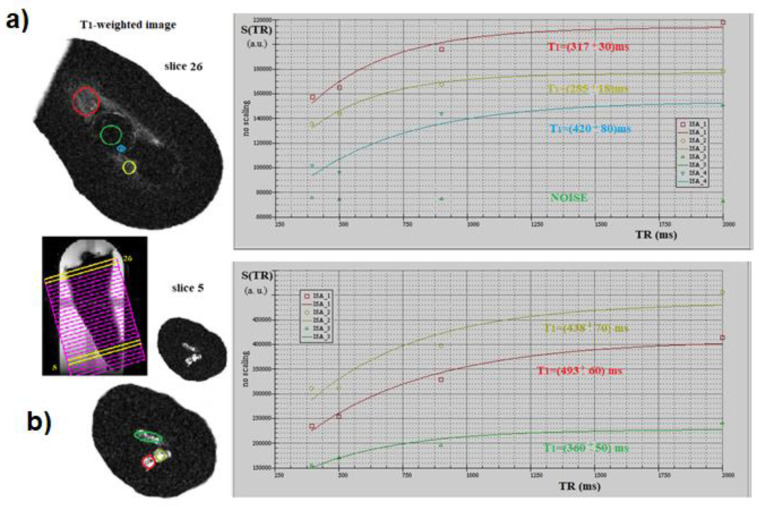
T1 quantification in different tooth ROIs. The green ROI in the selected Gutta-percha area shows no signal, i.e., noise (**a**). Other selected zones of the tooth (red, yellow and blue ROIs) are characterized by different T1 values. Specifically, hyperintense voxels (bright white) highlighted in blue ROI of (**a**) and the three ROIs in (**b**), indicating incomplete filling, are characterized by a higher T1 value if compared to the tertiary dentin (red and yellow ROI) present in caption (**a**). In (**b**), the apical area of the tooth is not completely sealed, since there is an appreciable amount of water around the Gutta-percha cone: this could be explained by the fact that the cold condensation technique used was unable to guarantee a three-dimensional effective sealing.

**Figure 5:**
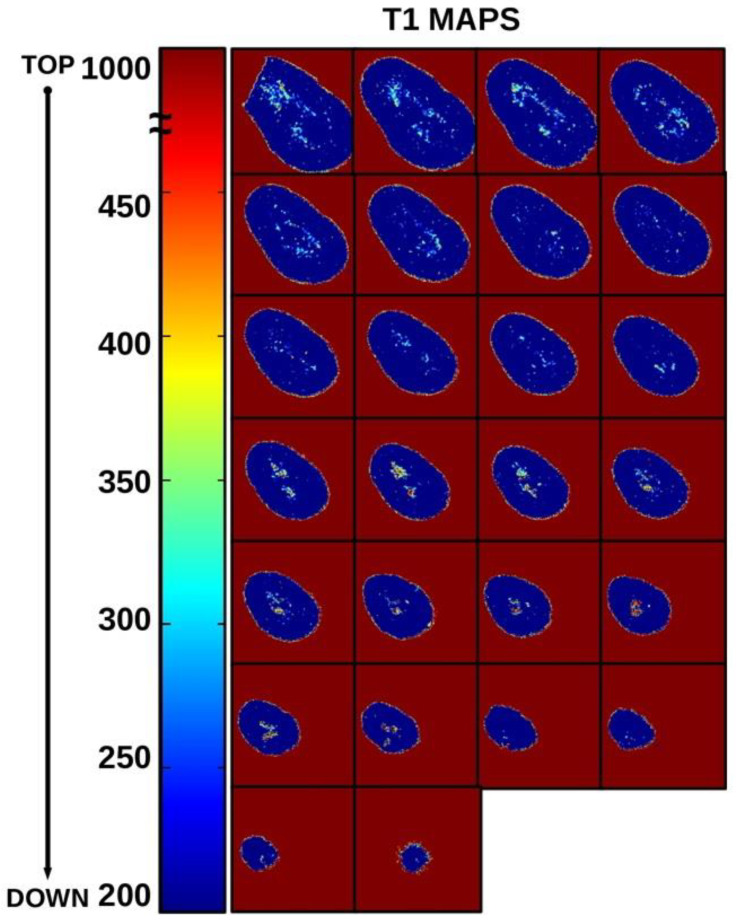
T1 map axial slices. Primary dentine is characterized by T1 values around 200 ms or less, and free water has T1 values more than 1000 ms. All voxels with T1 values ranging between 200 and 1000 ms indicate zones of tertiary dentin and/or incomplete filling zones. Comparing the images in this figure with those shown in Figure 1, it is possible to highlight the complementary information provided by T1 mMRI maps and CBCT images.

**Figure 6:**
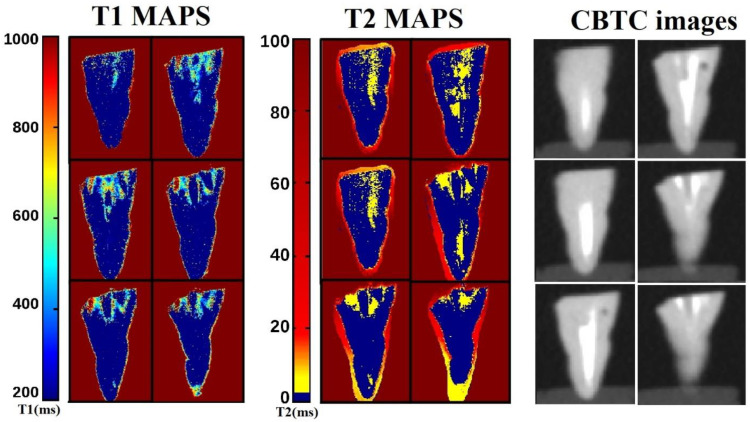
T1, T2 maps and CBCT images obtained from the same tooth slices showing the different and complementary image contrast assessed by mMRI maps and CBCT images. Water is present inside the canal, both in the coronal, middle and apical thirds, showing an incomplete three-dimensional filling and consequently fluid leakage. By considering the T1 and T2 values, in the last apical 3 mm, it is possible to evidence the presence of some inorganic tissue and water (light blue and yellow areas).

**Figure 7:**
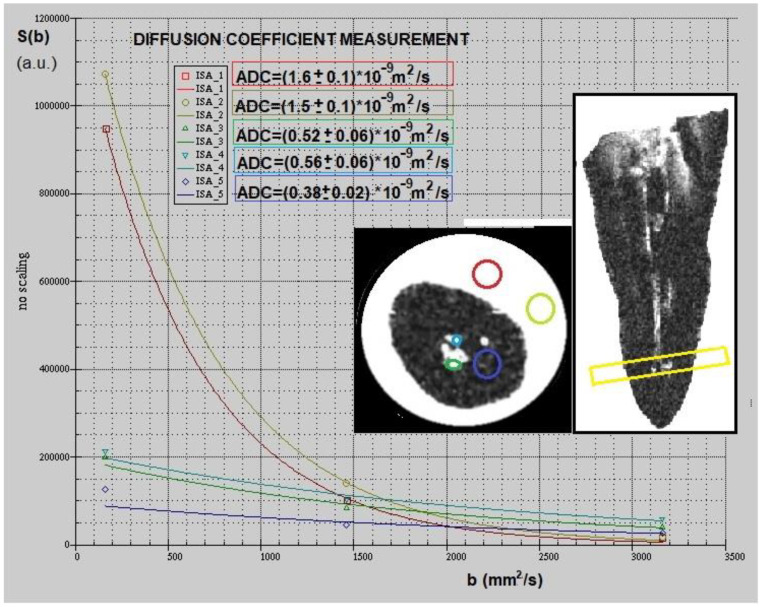
Diffusion weighted images of the tooth and fits function to experimental data from which ADC values were extracted and quantified in each region highlighted with different colors. The yellow slice in the parasagittal section represents the beginning of the apical third: in this specific case, this area is not sealed by Gutta-percha. In the axial image it is indeed possible to distinguish the terminal tip of the Gutta-percha cone (black circle between blue and light blue ROIs), the dentin (blue circle) and the aqueous infiltrate with some debris (light blue and green ROIs).

**Figure 8:**
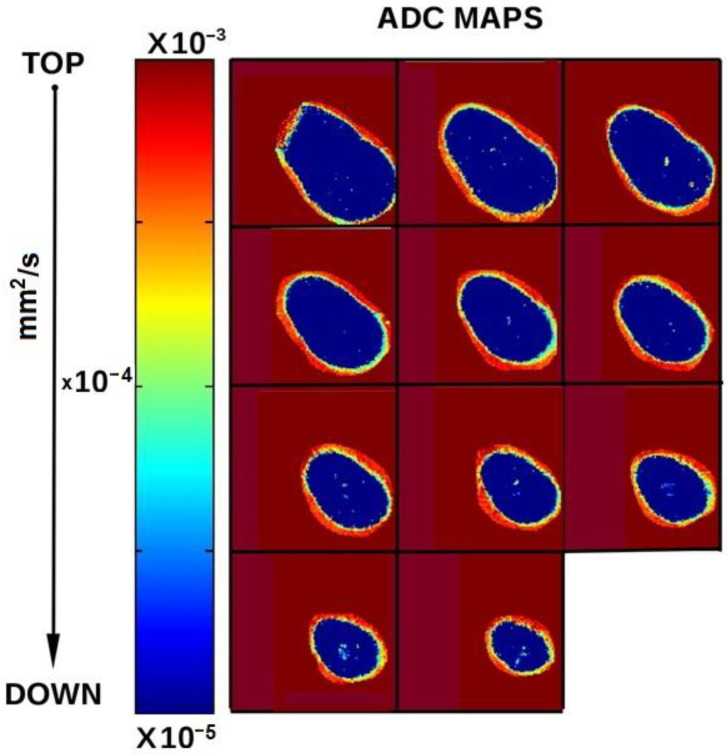
Axial slices of the ADC maps. Pixels with a color different from deep blue indicate the presence of water in pores and spaces, in a larger amount than in normal dentine, representing inadequate root canal filled zones.

Multiparametric mMRI provided complementary results: in Figure 2, a transversal view of T2-weighted image is displayed together with the fitting procedure, showing different T2 values in different tooth regions. In the T2-weighted image, obtained at TE = 2.3 ms, voids and underfilled canal are clearly visible as hyperintense signal (white voxels) while Gutta-percha provides no signal, or noise, since this material does not contain moving liquid-like protons. On the other hand, porous dentin provides a low intensity MRI signal due to the rapid relaxation of the mobile protons which are confined in microporous matrix. These water protons are affected by the rapid signal dephasing due to the magnetic susceptibility difference between the liquid in the pores and the solid matrix [13,14]. The image displayed in Figure 2 shows that larger cavities are characterized by longer T2s. The water within which the tooth is inserted shows the contours of the Gutta-percha, or the cement–dentin border in the coronal third (marked with red rows). The large hyperintense signal shows incomplete apical filling at different degrees that correspond at different T2 values (red and yellow ROI). Axial T2-weighted images are displayed in Figure 3, whereas T1-weighted images with T1 quantification in different tooth ROIs and T1 maps are reported in Figure 4 and Figure 5. T1, T2 maps and CBCT images obtained from the same tooth slices are showed in Figure 6 and the ADC results are reported in Figure 7 and Figure 8.

## 4. Discussion

Magnetic resonance imaging (MRI) is a common imaging modality used in various medical fields as well as in material science. Among medical applications, MRI in dentistry is spreading since detection of neoplasms, inflammatory and pathologic conditions of the mouth floor, tongue, salivary glands and TMJ are even more often diagnosed by MRI [17,18,19,20].

While hard tissue can be more easily visualized by traditional radiology, MRI is not the preferred choice in dentistry since the hard structures cannot be visualized clearly and appear as no-signal zones. As opposed to hard dental tissues, soft dental tissues have higher content of water and consequently, a much longer T2 relaxation times which could provide a high-quality MRI signal [10]. Based on these concepts, it was proposed that a high MRI signal of soft dental tissues could enable a high-resolution imaging of a dental pulp anatomy. An initial attempt in this direction was done by Lockhart et al. [21], who adopted a strong 9.4 T magnetic field to obtain MR images of the pulp chamber in vitro and to visualize the tooth outline. Tanasiewicz demonstrated the use of the 3D spin-echo MR imaging technique as a tool to visualize the inner space in the root canal system during the prosthodontic procedure for post preparation [22]. Susterstic and Sersa [23] demonstrated that MR microscopy could provide very accurate 3D visualization of dental pulp anatomy in vitro. Moreover, Tymofiyeva et al. [24] used clinical MRI scanners to visualize dental pulp in vivo and a comparative study by Gaudino et al. [8] using MRI and X-ray CT techniques showed that MRI, being capable of better characterizing soft tissues, could be applied to the detection of dental inflammatory or neoplastic pathologies at an early stage. In the present study, high resolution MRI was used to detect quality of canal obturation. As previously stated, when a void is shown by a radiographic or CBCT image, clinicians have no clue whether this area is simply a void due to inadequate root canal filling procedure or an area filled with left organic debris due to inadequate cleaning and shaping procedures. The hypothesis was that mMRI could be able to detect unfilled void and to distinguish whether a void was filled or not with soft tissues or debris, by determining different water contents, relaxation times and diffusion coefficients.

Observing the axial T2-weighted images along the entire tooth displayed in Figure 3, it is possible to investigate more in-depth the failure of the missed treatment. In Figure 3, in slice 24 of the coronal third, the contours of two Gutta-percha cones are visible and the less dense dentine (tertiary dentin) is appreciable in a large area around the cones. In slice 20 of the coronal third, the tertiary dentin space results reduced, the space filled by water was smaller, and the liquid minimally surrounded the Gutta-percha cone. In slice 16 of the middle third, the Gutta-percha cone showed a stricter adhesion to the walls of the endodontic canal with none or only a few areas with aqueous content (whiter voxels). Finally, at slice 4 of the apical third, it is possible to observe the presence of a well-marked aqueous infiltrate, able to clearly delineate the geometry of the apical delta. The presence of this large area (visible from slice 15 to slice 4) characterized by a high-water content, certifies the absence of filling material along the last apical 3.3 mm.

In Figure 4, the T1 quantification in different tooth ROIs is displayed. The green ROI in Figure 4a, selected in the Gutta-percha area, showed no signal or noise. Other selected zones (red, yellow and blue ROIs) are characterized by different T1 values: hyperintense voxels (bright white) highlighted in blue ROI of Figure 4a,b, indicating incomplete filling, are characterized by a higher T1 value if compared to tertiary dentin (red and yellow ROI in Figure 4a).

In Figure 5, T1-map axial slices are displayed: primary dentine is characterized by T1 values of ~200 ms or less, free water has T1 values higher than 1000 ms. All voxels with T1 values between the 200 and 1000 ms range are considered zones of tertiary dentin and/or incomplete filling zones.

In Figure 6, T1, T2 maps and CBCT images obtained from the same tooth slices are displayed to show the different and complementary image contrast assessed: the apical foramen is clearly visualized and appears to be positioned on the lingual surface of the root (5th and 6th rectangles of each map). Water is present inside the canal, both in the coronal, middle and apical third, showing an incomplete three-dimensional filling and the consequent fluid leakage. By considering the T1 and T2 values measured in the last apical 3 mm, the presence of some inorganic tissue and water, evidenced and confirmed by brighter areas, is appreciable.

In Figure 7, a different image contrast provided by the ADC weighted images is displayed. The contrast is based on the more or less restricted diffusion of water inside the tooth structures. In diffusion-weighted images, the more hyperintense voxels represent free water diffusion. When water diffusion is obstructed or restricted in small spaces, a darker gradation of the voxel becomes visible. Red and light green ROIs in Figure 7 show where free bulk water diffusion occurs. Therefore, the associated voxels are hyperintense and the diffusion coefficient ADC extracted by the fitting procedure of Equation (3) to data is equal to (1.5 ± 0.1) × 10^−3^ mm^2^/s. Conversely, water in dentine is characterized by ADC = (3.8 ± 0.2) × 10^−4^ mm^2^/s (violet ROI) and water diffusing in spaces larger than dentine pores, assumes ADC values greater than primary dentin. In the axial image it is indeed possible to distinguish the terminal tip of the Gutta-percha cone and the aqueous infiltrate with the presence of some debris (blue and green ROIs) which are characterized by a higher ADC value than healthy dentine. 

In Figure 8, axial slices obtained from ADC maps are reported: it is possible to evidence the presence of water inside pores and spaces which are larger than those present in normal dentine. 

Multiparametric mMRI results reported in this work indicate that the set of T1, T2, ADC values and the amplitude and geometry of the voids allow us to understand the nature of the unfilled voids. As shown in Figure 6, T2 maps immediately indicate the presence of voids and T1 maps indicate a variety of T1 values in tooth voids selecting different bulk water (Figure 5 and Figure 6). In the presence of organic debris, the relaxation time T1 assumes higher values than dentin but lower than voids filled mainly with water (Figure 4). As the residuals in the voids increase, the ADC value decreases. In fact, the ADC parameter quantifies the molecular diffusion [25]: molecules larger and heavier than water (such as organic molecules of debris) are characterized by slower dynamics and, therefore, they provide ADC values lower than the ADC values of bulk water (about 2 × 10^−9^ m^2^/s at room temperature) [26]. ADC is also reduced if compared to bulk water value, due to restricted diffusion. Restricted diffusion occurs when the diffusion length l_D_, (the characteristic length probed by diffusing molecules) at the diffusion time t, l_D_ = (6*ADC*t) which is about 40μs for free water, is less than the diffusion length of pores or voids in which water diffuses [27]. In this work, ADC of restricted diffusion water in normal dentin is about (3.8 ± 0.2) × 10^−10^ m^2^/s (Figure 7) where dentine pores (microtubules’ cross-sections and their interconnections) are characterized by a mean diameter of about 1–2 μm. Water-filled larger pores are characterized by higher values of ADC compared to that of normal dentine (Figure 8).

Based on these findings we can affirm that mMRI has a potential to become a valuable non-invasive device to detect and analyze pulp tissue inside the endodontic space, and consequently assess quality of shaping, cleaning and filling procedures. To date, pulp anatomy and endodontic procedures have been non-invasively investigated only from 2D or 3D tooth radiographs, in which soft dental tissues are presented only indirectly as signal voids (empty spaces) inside the tooth. MRI is the only non-invasive technique that enables visualization of soft dental tissues, differentiates, and possibly quantifies the soft tissue left inside the endodontic space after the procedures, both in vitro (as shown in the present study) and potentially in vivo [10,28].

The use of micro-CT could allow a better visualization and comparison of the data obtained by mMRI. The use of CBCT in this study represents a weakness since the resolution is lower and the quality of the image is poorer, but it is more affordable for clinical usage than micro-CT, which is only available in vitro. The use of mMRI for clinical purposes in dentistry is still a challenging objective and the use of proper devices such as intraoral radio frequency probes is mandatory due to the manner in which they obtain high quality and comprehensive images for the dental practitioner [29,30]. However, some recent works [31,32] suggest that mMRI in endodontics will have a rapid development in the near future.

The limitations of this study concern the use of only one tooth. However, in this preliminary work we would like to show in the same tooth the different high resolution parametric maps that can be obtained with a multi-parametric mMRI approach and compare them with the results currently offered by techniques using ionizing radiation. Although our results are obtained on a single tooth, in this paper we report for the first time (to our knowledge) T2, T1 and ADC maps obtained on the dentinal tissue with a linear resolution of about 30 microns. We believe that the information contained in our manuscript may be a very useful starting point for developing both future ex vivo and in vivo diagnostic protocols useful in endodontics.

## 5. Conclusions

Although the radiographic examination will still be the diagnostic method of choice in endodontics, mMRI may also take its place in future as an adjunct diagnostic device, due to its several advantages: it does not involve ionizing radiation and it can differentiate tissues, vessels and nerves in regenerative techniques.

In this paper, we showed in vitro at the high magnetic field and high-performance magnetic field gradient the high diagnostic potential of multiparametric mMRI in endodontics for improvement in the detection and identification of small areas in incompletely cleaned reticular canals that are not clearly highlighted with CBCT. In particular, the multiparametric mMRI approach allows for the identification of the nature of what is left inside “voids” in root canals that currently can be assessed only by histology, which is a destructive methodology and cannot be used clinically.

Although it is not currently possible to use mMRI (i.e., NMR images with resolutions around tens of microns) in vivo with clinical scanners, the rapid technological development to which NMR instrumentation is subjected will most likely allow the use of mMRI in the endodontic field in the near future. However, it is currently possible to exploit mMRI technology in vitro, to refine and develop techniques and procedures (or even test new endodontic materials) for endodontics procedure optimization.
